# Host factors determine the evolution of infection with *Staphylococcus aureus* to gangrenous mastitis in goats

**DOI:** 10.1186/s13567-018-0564-4

**Published:** 2018-07-25

**Authors:** Pascal Rainard, Christophe Gitton, Thierry Chaumeil, Thierry Fassier, Christophe Huau, Mickael Riou, Gwenola Tosser-Klopp, Zuzana Krupova, Anne Chaize, Florence B. Gilbert, Rachel Rupp, Patrice Martin

**Affiliations:** 1 ISP, INRA, UMR 1282, Université Tours, 37380 Nouzilly, France; 2 PFIE, INRA, UE 1277, 37380 Nouzilly, France; 3 INRA, UE 0332, Bourges-La Sapinière, 18390 Osmoy, France; 4 GenPhySE, INRA, UMR 1388, Université de Toulouse, 31326 Castanet-Tolosan, France; 5 GABI, INRA, UMR 1313, Université Paris Saclay, 78350 Jouy-en-Josas, France; 6 EXCILONE, 78990 Elancourt, France

## Abstract

*Staphylococcus aureus* is the major cause of very severe mastitis of dairy goats. The initial objective of our study was to fine-tune an experimental model of infection of the goat mammary gland with two strains of *S. aureus* and two lines of goats (low and high somatic cell score lines). Following the challenge, the 10 infected goats divided in two clear-cut severity groups, independently of the *S. aureus* strain and the goat line. Five goats developed very severe mastitis (of which four were gangrenous) characterized by uncontrolled infection (UI group), whereas the other five kept the infection under control (CI group). The outcome of the infection was determined by 18 h post-infection (hpi), as heralded by the bacterial milk concentration at 18 hpi: more than 10^7^/mL in the UI group, about 10^6^/mL in the CI group. Leukocyte recruitment and composition did not differ between the groups, but the phagocytic killing at 18 hpi efficiency did. Contributing factors involved milk concentrations of α-toxin and LukMF′ leukotoxin, but not early expression of the genes encoding the pentraxin PTX3, the cytokines IL-1α and IL-1β, and the chemokines IL-8 and CCL5. Concentrations of TNF-α, IFN-γ, IL-17A, and IL-22 rose sharply in the milk of UI goats when infection was out of control. The results indicate that defenses mobilized by the mammary gland at an early stage of infection were essential to prevent staphylococci from reaching critical concentrations. Staphylococcal exotoxin production appeared to be a consequent event inducing the evolution to gangrenous mastitis.

**Electronic supplementary material:**

The online version of this article (10.1186/s13567-018-0564-4) contains supplementary material, which is available to authorized users.

## Introduction

Breeding programs for mastitis resistance in dairy cattle, sheep and goat make use of the somatic cell score (SCS) as a selection criterion [1–3]. Divergent SCS-based selection experiments in sheep and goats have shown that the low SCS groups were more resistant to mastitis under field conditions [4, 5]. This is particularly true of infections with staphylococci, which are responsible for the majority of intramammary infections of dairy small ruminants [6, 7]. Experimentally-induced mastitis of two lines of ewes selected on the basis of high/low SCS with *Staphylococcus aureus*, the agent of the most severe forms of staphylococcal mastitis, have shown some line-associated differences in response to infection [8]. Studies on SCS-based divergent selection of animals is of great help to settle the issue of the selection for low somatic cell count (SCC) in relation to mastitis susceptibility [9].

The objective of the study was to expand our knowledge of the course of *S. aureus* infection of the caprine mammary gland in order to investigate the responses of two divergent lines of goats selected for high and low SCS. Previous experiments with goats, which aimed at identifying differences in the immune response to experimental infections with *S. aureus* had not revealed obvious factors distinguishing animals selected on the basis of the SCS [10]. It thus appeared necessary to adapt the experimental model to favor the disclosure of differences between groups. A small scale experiment involving ten goats was designed with a view to fine-tuning the model of experimental infection. A preliminary step was to select infecting strains that have the potential to induce mammary gland infections neither too mild nor too severe, but of intermediate severity fit to uncover different responses to infections. To this end, we compared the response to two *S. aureus* strains representative of caprine mastitis-causing strains of anticipated different virulence, and we monitored the course of infection over a week. One strain had been used previously to experimentally infect goats, causing mild to severe mastitis [11]. Another strain producing less leukotoxin was included in the experiment. This strain was deemed less aggressive because the bi-component leukotoxin LukMF′ is likely to contribute to the severity of *S. aureus* mastitis in dairy ruminants [11, 12]. Unexpectedly, five goats developed very severe mastitis, four of which gangrenous, and the severity was independent of the goat selection line or the challenge strain. This experiment gave us the opportunity to explore the pathophysiology of severe (gangrenous) mastitis, and we took advantage of the clear-cut delineation of the two severity groups to analyze the events that segregated the goats and determined the outcome of infection. Although we already know a great deal about *S. aureus* mastitis pathogenesis, there are still gaps in our understanding of the major mechanisms that determine the severity of the infection [13].

## Materials and methods

### Animals, experimental scheme and sample collection

Ten goats of Alpine breed from divergent selection for extreme breeding values for the somatic cell score (four goats from the low SCS line and six from the high SCS line) were used. These goats were produced and reared at the INRA herd of La Sapinière (Bourges, France) as described [5]. A previous study had shown that low SCS line goats displayed lower prevalence of infected udders and thus higher mastitis resistance than high SCS line goats when characterized in conventional breeding condition [5]. The ten selected goats aged 14 months were in their first lactation, had healthy uninfected mammary glands, and arrived at the INRA Experimental Infection Platform (PFIE, Centre de Recherche Val de Loire, Nouzilly, France) when they were 1–2 months in lactation for a 2-week acclimatization before infectious challenge. A small bolus (Small Bolus^®^, Médria Elevage, Chateaubourg, France) recording telemetric measurements of intraruminal temperature every 30 min was inserted into the rumen of each goat at their arrival at the experimental facility. At time of challenge, the goats weighed 30–45 kg (mean 38.5 kg) and produced about 3 kg milk/day. They were machine milked twice a day and fed a ration of hay and concentrate. They had access to water ad libitum.

Before the challenge, the udder-half to be inoculated was checked for the absence of intramammary infection by performing bacteriological analysis and SCC measurement on foremilk samples (Fossomatic model 90, Foss Electric, Hillerod, Denmark). Inoculated glands were free of infection and shed less than 100 000 cells/mL milk. After inoculation into one udder-half, the goats were monitored for a week before slaughter. The goats were not separated after inoculation and shared the same milking equipment.

### Intramammary challenge with *S. aureus*

Two strains of *S. aureus* were used in this study. Strain Ch122, initially isolated from the milk of a mastitic goat, had been used previously in a goat mastitis experiment [11]. This strain produces substantial amounts of α-toxin and LukMF′ leukotoxin when cultured overnight in brain heart infusion broth (about 25 and 3.3 µg/mL, respectively), and exhibits alpha and strong beta hemolysis on sheep blood agar plates. Strain Ch204, which was also isolated from goat milk (subclinical mastitis), exhibits alpha and beta hemolysis on sheep blood agar plates but produces somehow less α-toxin and LukMF′ leukotoxin (about 12.5 µg/mL and 650 ng/mL, respectively). The two strains were kept at −70 °C in BHI supplemented with 10% dimethylsulfoxide (Sigma). Before use, BHI broth was inoculated with frozen bacteria and incubated overnight at 37 °C. Purity of the culture was checked by plating on a sheep blood agar plate. To prepare the inoculum, a subculture was made in BHI for 4 h at 37 °C from an overnight BHI culture. Bacteria were then centrifuged (1400 × *g* for 5 min at 4 °C), washed twice with Dulbecco’s phosphate buffered saline (DPBS), and adjusted (OD at 600 nm) to the concentration of 1000 CFU/mL in DPBS. The inoculum was kept on ice and used within 2 h of preparation. Retrospective CFU checking by plating on trypticase soy agar (TSA) plates yielded 700 CFU/mL for strain Ch122 and 1020 CFU/mL for strain Ch204. Just before inoculation, the goats were milked, and the teat ends were carefully disinfected by swabbing with 70% ethanol. Inoculation was carried out through the teat canal of the right udder-half by injection of 1 mL of the bacterial suspension with a blunt-ended sterile cannula fitted with a disposable 1 mL syringe. Strain Ch122 was inoculated to goats #1, 2, 4, 7 and 8, strain Ch204 to goats #3, 5, 6, 9, and 10. The first milkings following inoculation were carried out 12 h and 18 h later.

### Clinical scoring and hematology

Systemic and local mammary signs were assessed through the trial period by two experienced experimenters according to the scoring scale described in Table 1. Clinical scoring was based on the measurement of body temperature (Small Bolus^®^), milk appearance, local udder signs (through palpation or mammary skin color), and systemic clinical signs. The clinical score was the additive result of the partial scores in these four categories. Moreover, weight loss was monitored by weighing the goats on the day of inoculation and 5 days later (survivors only).

**Table 1:** **Clinical scoring of mastitis**

Notation	Temperature	Milk appearance	Udder signs	Systemic signs
0	Normal	Normal	Normal	Normal
1	39–40 °C	Small clots	Swollen	Feverish
2	40–42 °C	Large fibrin clots	Sore, hardened	Slightly depressed
3		Serous or bloody	Discolored	Deeply depressed
4			Bluish	Recumbent

Blood cells were counted with a MS9-5 Hematology Counter^®^ (digital automatic hematology analyzer, Melet Schloesing Laboratories, France). Twenty-nine parameters were analysed, which characterized three categories of blood cells: (1) total white blood cells (lymphocytes, monocytes; neutrophils; eosinophils; basophils and others white cells); (2) red blood cells and (3) platelets. The white blood cell and neutrophil counts were followed to monitor the inflammatory response after bacterial infection.

### Processing and examination of milk and blood samples

Foremilk samples from the challenged quarters were taken aseptically at intervals, every 6 h for the first 24 h, then at each milking. Portions were used to determine the SCC using a cell counter (Fossomatic model 90, Foss Food Technology, Hillerod, Denmark), to assess the occurrence of bacteria within and without phagocytes, and to quantify bacterial counts. The remaining milk was centrifuged (1000 ×* g* for 30 min at 4 °C), the fat layer removed, and the skim milk stored at −20 °C for subsequent analyte measurements.

Bacterial clusters and phagocytes in the milk of infected glands were examined by preparing cytospin slides by cytocentrifuging (Shandon Southern centrifuge, Pittsburgh, PA, USA) 100 µL of milk plus 30 µL of fetal calf serum on glass slides that were stained with May–Grünwald–Giemsa stain. For determination of *S. aureus* bacterial counts, series of tenfold milk dilutions were performed by mixing 200 µL of milk with 1.8 mL of PBS plus 0.5% Tween 80 and vigorous mixing, then 100 µL of the dilution with 900 µL of PBS-Tween 80. Appropriate dilutions were plated on TSA, incubated for 24 h at 37 °C, and colonies enumerated.

### Staphylococcal toxins and antibody quantification

The bi-component leukotoxin LukMF′ was quantified in milk, and antibodies to LukM were quantified in blood serum by Enzyme-Linked Immunosorbent assay (ELISA) as described [11]. Sera were titrated with reference to the serum of a goat immunized with purified LukM, which was arbitrarily given a titer of 1000 units. The α-toxin was purified as previously described [14], and antibodies to α-toxin were measured by indirect ELISA [15]. Sera were titrated with reference to a pool of goat sera that was arbitrarily given a titer of 200 units. Alpha-toxin concentrations were determined by competitive ELISA as described [16].

### Assay of phagocytic bactericidal activity of whole fresh milk

Milk from infected quarters was taken at 18 hpi with aseptic precautions. Within 2 h of collection, 1 mL of milk was incubated for 1 h at 37 °C under end-over-end agitation after addition of 100 µL of *S. aureus* (Ch204) suspension (10^7^ CFU/mL). Strain Ch204 only was used because preliminary experiments had indicated that the two strains Ch122 and Ch204 did not show noticeable different opsonic requirements and resistance to killing by neutrophils. In parallel, a control tube containing the same phagocytic mixture was incubated at rest. An aliquot from each phagocytic mixture was serially diluted in PBS + 0.5% Tween 80 before plating on TSA. After an overnight incubation at 37 °C, colony-forming units of each culture plate were enumerated. Bacterial survival percentage was calculated from the reduction in bacterial numbers in agitated tubes compared to bacterial numbers in tubes kept at rest.

### ELISA for chemokine and cytokine quantification

A sandwich ELISA for the chemokine IL-8 (also known as CXCL8) was performed by using 1 µg/mL affinity-purified rabbit antibodies to bovine IL-8 (Kingfisher Biotech) as capture antibody, biotinylated affinity-purified rabbit antibodies to bovine IL-8 C-terminal peptide as detection antibody, and recombinant bovine IL-8 as standard as described [17]. The ELISAs for CXCL3 or IL-17A were performed as described with antibodies to bovine CXCL3 or IL-17A, which cross-react with caprine CXCL3 or IL-17A, respectively, due to high amino-acid sequence identity [17, 18]. A commercially available kit for bovine IFN-γ was used, with cross-reaction for caprine IFN-γ established by the manufacturer (Mabtech AB, Nacka Strand, Sweden). The ELISA for TNF-α was performed as described [19], with reagents developed for bovine cytokines, which cross-react with TNF-α from small ruminants [20]. ELISA to IL-22 was performed as described [21], with antibodies to bovine IL-22 that were deemed to cross-react due to high amino-acid sequence identity (91%) of the bovine and caprine molecules.

### Real time quantitative PCR on RNA extracted from milk fat globules (MFG)

Fifty milliliters milk samples were centrifuged at 2000 ×* g* for 10 min at 4 °C. Using a sterile spatula, the supernatant fat layer was transferred to a 50 mL Falcon tube. Then, 1 g of fat was weighed in a 15 mL Falcon tube, 3 mL TRIzol™ LS (Invitrogen, Life Technologies) were added, and the tube was vortexed vigorously prior storing the samples at −80 °C. Total RNA was then isolated, using the manufacturer protocol, essentially as previously described [22]. Concentration, purity and integrity of total RNA extracted from MFG were assessed with two devices: purity of the RNA was evaluated by using a NanoDrop ND-1000 spectrophotometer (ThermoFisher Scientific). Concentration and integrity were determined using the Agilent BioAnalyzer 2100 system (Agilent Technologies) with RNA 6000 Nano LabChip Kit. Quality was evaluated using the RNA Integrity Number (RIN) value [23]. First strand cDNA was then synthesized from 1 ng of total RNA, primed with oligo(dT)20 and random primers (1:1, v/v) using the Superscript III reverse transcriptase (Invitrogen, Life Technologies) according to the manufacturer’s instructions. Single-stranded cDNAs thus obtained were quantified using the Agilent BioAnalyzer 2100, and then stored at −20 °C.

To determine the expression of a set of selected genes, primers were designed using the Primer Express™ Software v3 (Applied Biosystems, ThermoFisher Scientific) using gene sequences (*Ccl5*, *Il1α* and *MIF*), from a caprine database (*Capra hircus*, ID 10731, NCBI), or using primers previously designed [24] (Table 2). All qPCR amplification systems were first validated as described [24]. qPCR was performed using the Power SYBR^®^ Green Master Mix (Life Technologies, ThermoFisher Scientific) and a QuantStudio™ 12K Flex Real-Time PCR System (Applied Biosystems, ThermoFisher Scientific). Each qPCR well contained 10 µL Master mix, 5 µL cDNA and 1.2 µL of each primer at 5 µM (300 nM final) and nuclease-free water for a final volume of 20 µL. Reactions were run in triplicate. The results generated by the QuantStudio™ 12K Flex Software v1.2.2 (Applied Biosystems, ThermoFisher Scientific) were then exported as “.eds” files and analyzed with the Standard Curve version 3.4 (ThermoFisher Cloud apps). Relative expression levels were calculated using the software qBase [25], using the reference genes *RPS24* and *PPIA*.

**Table 2:** **Primers used for the qPCR carried out with the RNA extracted from MFG**

Genes	Primers	Sequence 5′–3′	Amplicon (bp)
*Ccl5*	Forward Reverse	ACC AGC AGC AAG TGC TCC AT CGC ACA CCT GAC GGT TCT T	86
*Cd68*	Forward Reverse	GAT CTG CTC TCC CTG AAG CTA CA CAT TGG GAC AAG AGA AAC TTG GT	79
*Csn1s2*	Forward Reverse	CTG GTT ATG GTT GGA CTG GAA AA AAC ATG CTG GTT GTA TGA AGT AAA GTG	76
*Il17a*	Forward Reverse	GCC CAC CTA CTG AGG ACA AG GCT GGA TGG TGA CAG AGT TC	246
*Il17f*	Forward Reverse	CACTCTGGAGGACCACATTG GAGTTCAGGGTCCTGTCTTC	216
*Il1a*	Forward Reverse	CTG AAG AAG AGA CGG TTG AG ATG CAT TCC TGG TGG ATG AC	164
*Il1b*	Forward Reverse	GAC AAC AAG ATT CCT GTG GCC TCT ACT TCC TCC AGA TGA AGT GT	101
Il8	Forward Reverse	TGA GAG TGG GCC ACA CTG C CAC AAC CTT CTG CAC CCA CTT	103
*MIF*	Forward Reverse	GCG CCT GCG ATT AGC CGC GTT CAT GTC GCA GAA	55
*PTX3*	Forward Reverse	CCG AGC TGT GCA GGG CT GCA CGC TTG CAA AAA TCT TCT T	101
*PPIA*	Forward Reverse	TGA CTT CAC ACG CCA TAA TGG T CAT CAT CAA ATT TCT CGC CAT AGA	62
*RPS24*	Forward Reverse	TGG TGGTGG CAA GAC AAC TG TTC TTC GCG TAA TCC AAG GAA	66

Gene names: C–C motif chemokine ligand 5 (CCL5); Alpha-S2-casein (CSN1S2); Macrophage migration inhibitory factor (MIF); Pentraxin 3 (PTX3); Peptidylprolyl isomerase A (PPIA); Ribosomal protein S24 (RPS24).

### Statistical analysis of data

Spearman’s rank correlations and Mann & Whitney test were calculated with GraphPad Prism^®^ 7.03 (GraphPad Software). The exploratory multivariate principal component analysis (PCA) was performed using the XLSTAT software (XLSTAT-Base^®^, Addinsoft) (Spearman’s correlation, distance biplot).

## Results

### Clinical recordings

**Figure 1:**
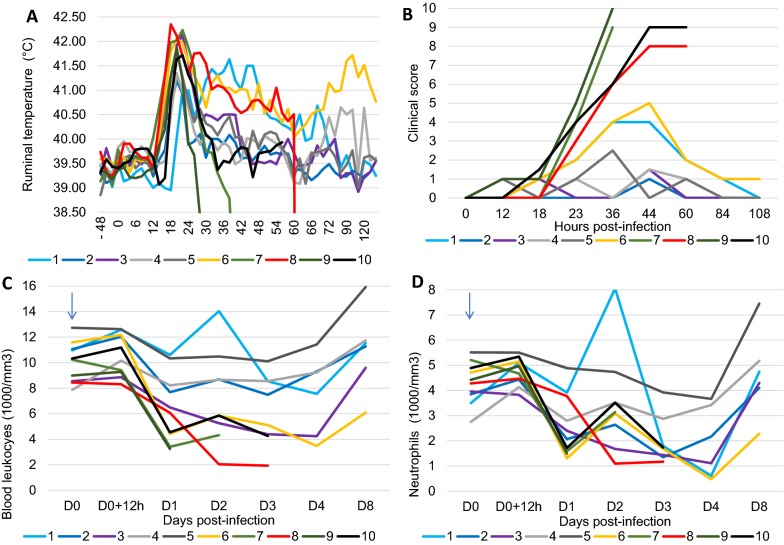
**Clinical and hematological parameters of infection. A** Intra-ruminal temperature following intramammary challenge with *S. aureus.* **B** Clinical score, including ruminal temperature, milk appearance and mammary and systemic signs. **C** Blood leucocyte count. **D** Blood neutrophil count. **A** and **B** X axis labels are hours post-infection (hpi), **C** and **D** X axis labels are days post-infection.

All goats developed a febrile reaction that peaked at about 24 hpi with values between 41.5 and 42 °C (Figure 1A). In most (seven out of ten) goats the temperature decreased rapidly in the following 24 h. The temperature fell below normal in three of the four goats that developed gangrenous mastitis concurrently with the appearance of severe systemic clinical signs (Figures 1A and B). Clinical scores distinguished three distinct trajectories: four goats reached very high scores, in relation with the appearance of gangrenous mastitis, four goats showed mild clinical signs, the other two being intermediary (Figure 1B).

Peracute mastitis of which five goats (no. 6–10) were affected, was characterized by systemic signs of disease. The body temperature rose to 41.5–42 °C, the goats were anorexic and profoundly depressed. They were often recumbent. The affected mammary gland was grossly swollen, hard and sore to touch. When gangrene developed, the skin color changed to a purplish red in patches, especially towards the base of the gland and around the teat. Milk secretion turned to a reddish exudate and finally milk production ceased. The goats that developed gangrenous mastitis (no. 7–10) were slaughtered on humane grounds soon after the signs of gangrene were definitely detected, except for the goat no. 9 who died overnight (by 30 hpi). The other five goats (no. 1–5) experienced a less severe form of mastitis that was characterized by local clinical signs and the occurrence of clots in milk. Nevertheless, they suffered from marked weight loss during the first 5 days of infection, which corresponded in magnitude with the clinical score (Table 3).

**Table 3:** **Weight (in kg) of goats before and on day 5 after infection**

Goats #	CCS line	*S. aureus* strain	Day 0	Day 5
1	CCS+	Ch122	45.870	41.380
2	CCS−	Ch122	40.250	38.560
3	CCS−	Ch204	44.190	41.640
4	CCS+	Ch122	35.340	32.030
5	CCS+	Ch204	32.480	31.900
6	CCS+	Ch204	36.650	31.090
7	CCS+	Ch122	29.990	ND
8	CCS−	Ch122	39.590	ND
9	CCS−	Ch204	39.120	ND
10	CCS+	Ch204	42.000	ND

ND: not determined.

There was no obvious relationship between the severity of infection and either the SCS selection group or the challenge *S. aureus* strain. It appeared that the most informative way to analyze the results was by considering individual trajectories, and by distinguishing two groups based on the severity and outcome of the disease. The group of the five goats (no. 1–5) who developed severe mastitis but controlled the infection was called the CI (controlled infection) group, and the group of five goats (no. 6–10) who suffered from out of control infections was called the UI (uncontrolled infection) group.

Hematological monitoring showed that all the goats experienced a drop in blood leukocyte counts during the first day post-inoculation (Figure 1C). The goats that developed the most severe mastitis exhibited a marked leukopenia. Leukocyte counts returned to normal by 8 days post-inoculation in the blood of the five goats of the CI group (Figure 1C). Neutrophil counts paralleled the total leukocyte counts, although with a worst downfall, revealing a severe neutropenia of most of the goats (Figure 1D).

### Local inflammatory response

**Figure 2:**
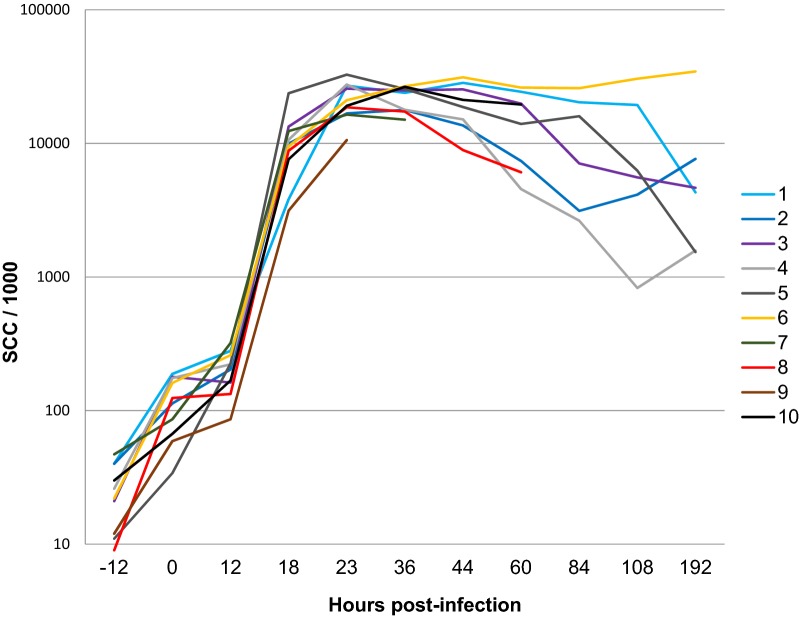
**Influx of leukocytes in the milk of infected glands.** The kinetics of SCC before (−12 h) and after inoculation of udder-halves of the ten goats at 0 h.

Milk SCC, which were initially low (less than 50 000/mL), increased slightly at time of challenge, most likely in relation with the sampling time of day, but did not increase substantially by 12 hpi (Figure 2). SCC rose sharply between 12 and 18 hpi in the milk of the inoculated gland of all goats (3 to 23 × 10^6^ cells/mL), and reached a plateau by 23 hpi (10 to 33 × 10^6^ cells/mL (Figure 2).

**Figure 3:**
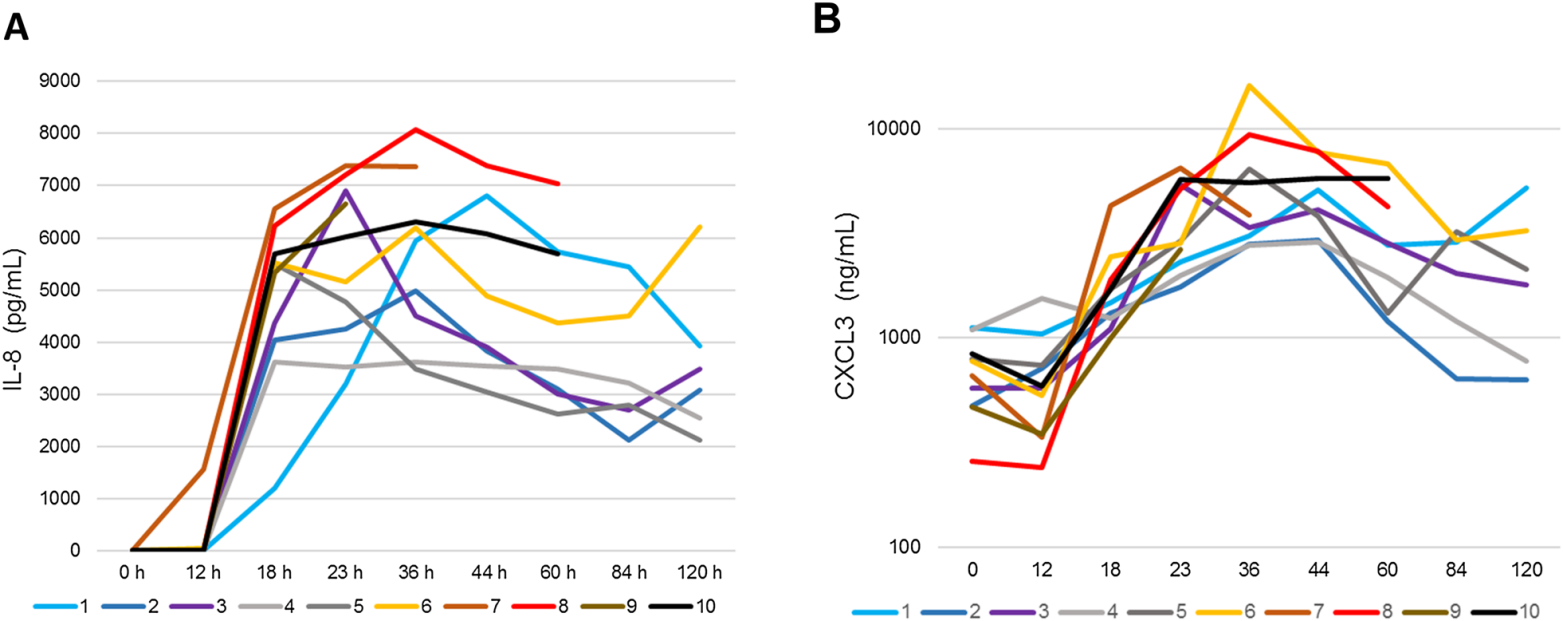
**Neutrophil-attracting chemokines.** Concentration of IL-8 (**A**) or CXCL3 (**B**) in milk of infected udder-halves after inoculation at 0 h.

The neutrophil-attracting chemokine IL-8 was not detected in milk before inoculation, and concentrations did not rise before 12 hpi except for one goat. Concentrations rose sharply between 12 and 18 hpi (Figure 3A). The concentrations of CXCL3, the constitutive milk chemokine, were already high and differed widely among goats before inoculation, from 257 to 1121 ng/mL (Figure 3B). Concentrations increased in response to infection only after 12 hpi.

**Figure 4:**
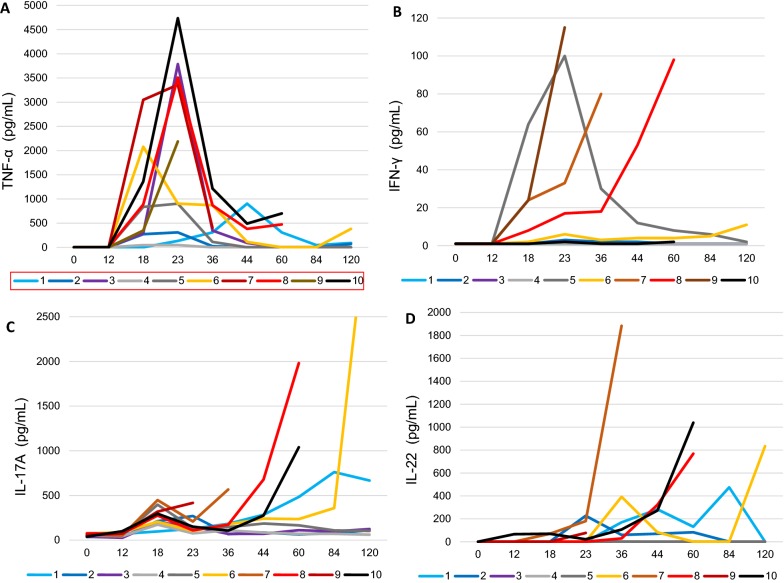
**Inflammatory and stimulatory cytokines.** Concentration of TNF-α (**A**), IFN-γ (**B**), IL-17A (**C**) and IL-22 (**D**) in milk of infected udder-halves after inoculation at 0 h. Labels of the X axis are hours post-infection.

Concentrations of the inflammatory cytokine TNF-α increased sharply in the milk of mainly the UI goats between 12 and 18 hpi, and reached a peak at 23 hpi (Figure 4A). There was a loose correlation between TNF-α concentrations at 18 hpi and the maximal clinical score (r = 0.616, *p* = 0.03). Concentrations of IFN-γ rose sharply only in the milk of three of the goats (no. 7, 8 and 9) that developed gangrenous mastitis, and one that did not (goat no. 5). Concentrations remained very low in the milk of the other goats (Figure 4B). Concentrations of IL-17A augmented in the milk of all inoculated mammary gland to reach a weak first peak (97–448 pg/mL) at 18 hpi (Figure 4C). Thereafter, concentrations decreased in the milk of goats that controlled the infection, except goat no. 1. By contrast, IL-17A rose sharply in the milk of the goats that developed gangrenous mastitis, reaching a maximum at their last sampling (Figure 4C). The same trend applied for IL-22 concentrations, except that there was no early concentration peak at 18 hpi (Figure 4D).

### Bacterial growth in infected glands

**Figure 5:**
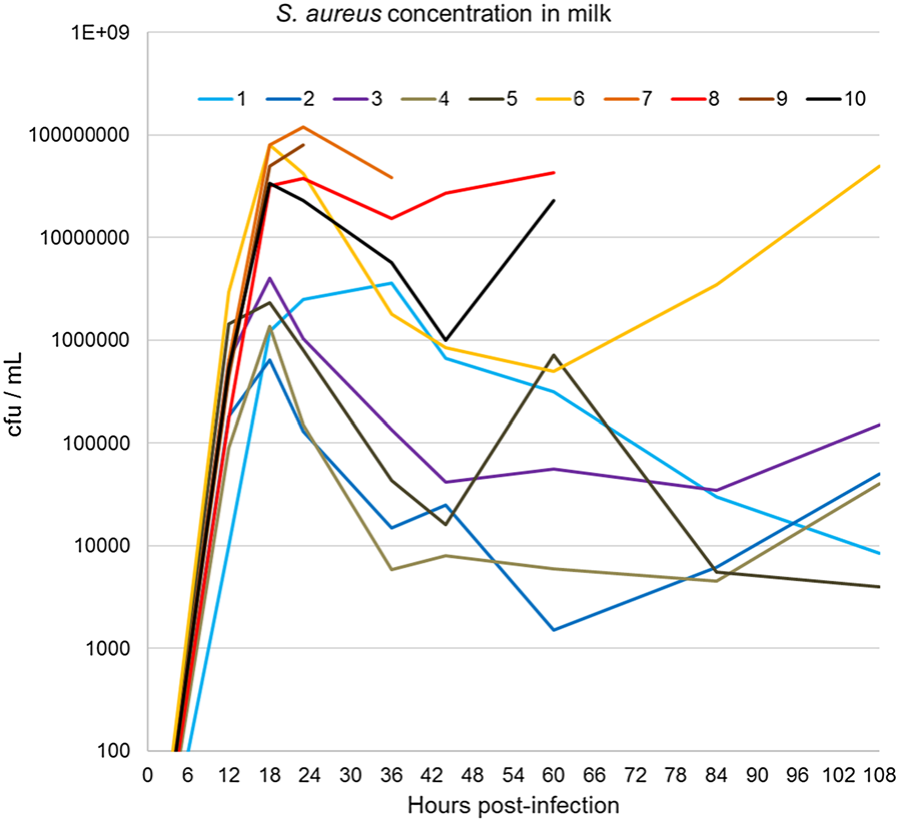
**Time course of** ***S. aureus*** **concentration in milk.** CFU were numbered by the plate count method. By 18 hpi, two groups of goats segregated on the basis of CFU concentrations (*p* < 0.01, Mann & Whitney test). The higher CFU group was referred to as the uncontrolled infection (UI) group. It comprised the four goats that developed gangrenous mastitis plus goat number 6, which labored to control the infection.

The monitoring of bacterial shedding in milk showed an unrestricted growth during the first 12 hpi in the milk of all goats (Figure 5). In all but one goat, bacterial concentrations exceeded 10^5^/mL, with a maximum of 2.92 × 10^6^/mL. Then the goats divided into two groups: those which controlled the bacterial load (goats no. 1–5), and those which did not (goats no. 6–10) (Figure 5). Within the former group, goat no. 1 was less efficient at reducing the bacterial load, which may be related to its tendency to have higher milk concentrations of TNF-α, IL-17A and IL-22 after 36 hpi (Figures 4 and 5). The latter group comprised the four goats which suffered from gangrenous mastitis, and goat no. 6 which somewhat controlled the infection for some time but finally lost ground, possibly in relation to its marked neutropenia on days 3 and 4 post-inoculation (Figure 1). We considered that the precautions taken at milkings were efficient to preclude the exchange of strains between goats, as no new infection by *S. aureus* occurred in the healthy non-inoculated half-udders during the monitoring period.

### Interactions between leukocytes and *S. aureus* in milk

**Figure 6:**
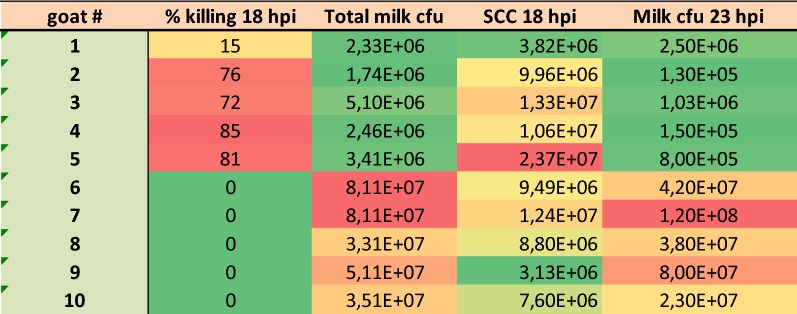
**Ex vivo phagocytosis (% killing) in milk samples taken at 18** **hpi.** Milk samples were incubated with gentle agitation at 37 °C, and % killing was calculated with reference to samples incubated at rest. Total milk CFU: the sum of in vivo grown bacteria in the sample plus 10^6^ in vitro grown bacteria. SCC 18 hpi: SCC in the 18 hpi milk samples. Milk CFU 23 hpi: concentration of viable bacteria in the samples taken 5 h after the samples under phagocytic test. Colors correspond to increasing values from green to red.

The assay of ex vivo phagocytosis performed with milk samples taken at 18 hpi showed clear-cut results: phagocytic killing was efficient in the milk of the CI group (although less so for goat no. 1), whereas there was no net killing in the milk of the UI group (Figure 6). The concentration of bacteria in milk delineated the dividing line between the two groups: bacterial concentrations differed by more than one log between the groups, and when bacterial concentrations exceeded 10^7^/mL, there was no net killing. Of note, there was no obvious difference between the two groups in terms of leukocyte concentrations in the milk samples (Figures 2 and 6), suggesting that the lack of phagocytic efficiency in the milk of UI goats was not due to insufficient cell concentration. In keeping with the importance of phagocytic killing efficiency for the control of infection, concentrations of bacteria in milk samples taken 5 h after the phagocytic samples (23 hpi) were significantly higher in the milk of UI goats than in the milk of CI goats (Figure 6; *p* < 0.001, Mann & Whitney test).

**Figure 7:**
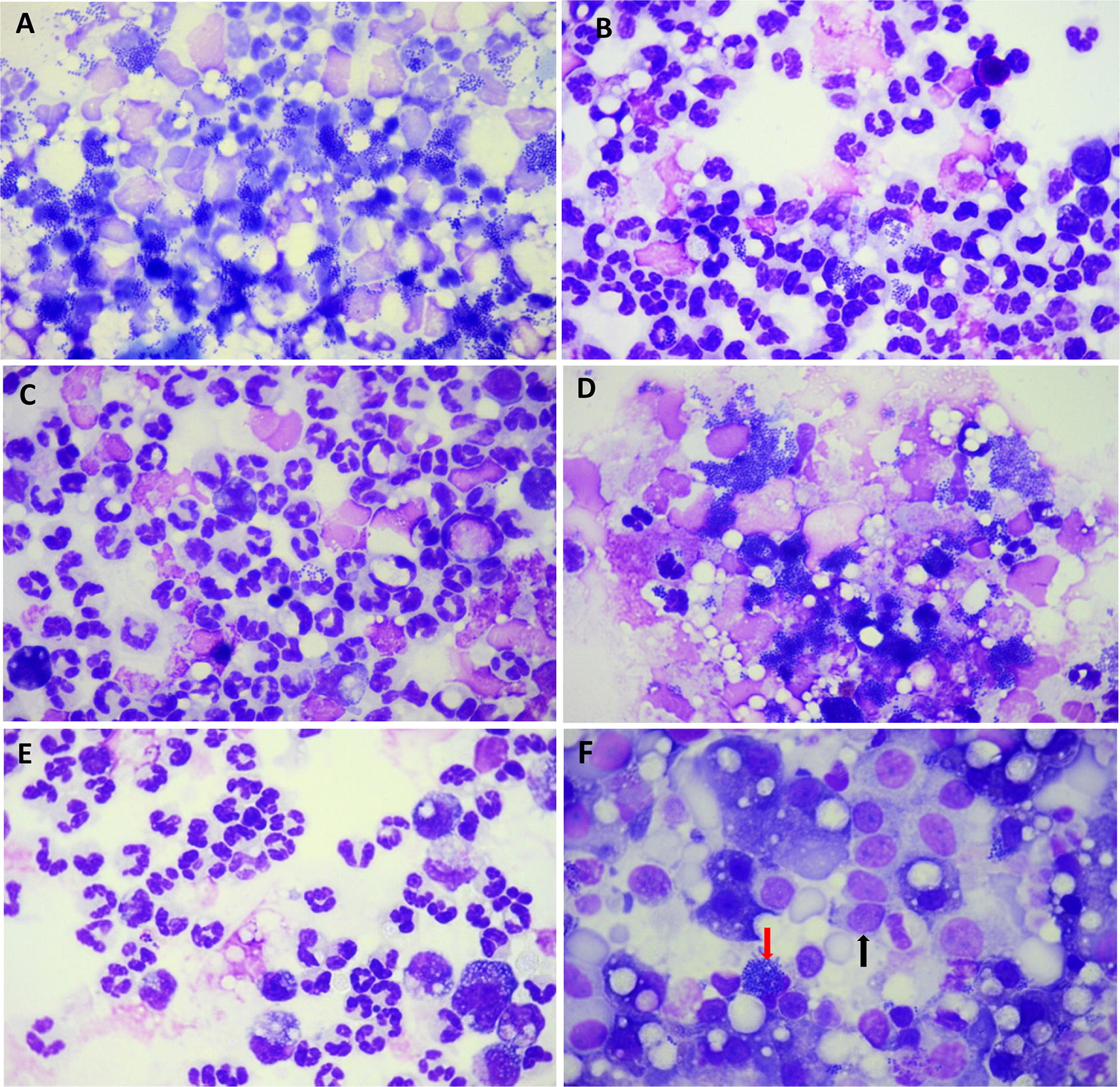
**Images of cytospin slides of milk samples taken from glands inoculated with** ***S. aureus***. Slides were stained with May–Grünwald–Giemsa. Representative slides are shown. **A** Proliferation of staphylococci in the milk of goat #7 at 18 hpi, with bacterial clusters next to cells with altered nucleus. **B** At 18 hpi, the milk of goat #5 shows images of phagocytosis, much less bacteria, and many neutrophils with recognizable nucleus. **C** Milk sample of goat #4 at 23 hpi showing mainly intact neutrophils. The arrow indicates a neutrophil with ingested staphylococci. **D** Milk sample of goat #9 taken at 23 hpi, about 7 h before death; clusters of staphylococci are visible, and cells are damaged. **E** Milk samples of goat #2 at 36 hpi, showing intact neutrophils and mononuclear leukocytes and very few staphylococci (arrow). **F** Milk samples of goat #7 at 36 hpi, showing clusters of staphylococci and cells whose nucleus has the characteristic aspect of neutrophils intoxicated with the leukotoxin LukMF′ (arrow).

The examination of cytospin slides (Figure 7) revealed very different pictures in relation to the severity and outcome of infection: the first difference was the occurrence of large bacterial clusters in the milk of the UI goats, compared to the milk of CI goats at 18 hpi, and an altered neutrophil appearance (Figures 7A and B). At 23 hpi, in the milk of the goats that reduced the bacterial burden, most neutrophils had a normal appearance and ingested staphylococci were visible (Figure 7C). By contrast, bacterial rafts were frequent in the milk of the goats that did not control the infection, and intact neutrophils were scarcely visible (Figure 7D). At 36 hpi, the divergent infection course of the CI and UI groups was confirmed, illustrated by intact neutrophils and few bacteria in the milk of CI goats (Figure 7E) and cells that had adopted the characteristic appearance of neutrophils intoxicated by the leukotoxin LukMF′, i.e. rounded and swollen nucleus [11], in the milk of UI goats (Figure 7F).

To assess if antibodies to the leukotoxin LukMF′ had contributed to the protection of CI goats, antibodies to LukM were quantified in serum samples taken at time of inoculation. Compared to an immune serum given 1000 antibody units, all goats had rather low titers (26–172 units), without obvious link with the outcome of infection (Additional file 1). As images of LukMF′-exposed neutrophils were seen in the milk of the UI goat, LukM concentrations in milk were determined and compared to the minimum concentration active on caprine neutrophils established in vitro, i.e. 18 ng/mL [26]. In this study, we supposed that the other component of the toxin (LukF′), was also produced at similar concentrations, as found in [11]. The leukotoxin component LukM was found at active concentrations only in the milk of the four goats that developed gangrenous mastitis (Additional file 1). The production of active concentrations of LukM preceded the appearance of the gangrene signs, and were already reached at 18 hpi in the milk of the four goats that developed gangrenous mastitis (Additional file 1). This may explain the phagocytic killing inefficiency manifested in the ex vivo assay, and why the concentration of phagocytes was not discriminant for the two groups of goats, since the Fossomatic determination does not distinguish live from dead leukocytes.

As α-toxin is considered an important player in the gangrenous form of mastitis, we also assessed the initial antitoxin titers and measured the concentration of the toxin in milk in the course of infection. Antitoxin titers were rather low and did not discriminate the two severity groups (Additional file 1). Sizeable concentrations of α-toxin were found in milk of the five UI goats, as soon as 18 hpi, and concentrations reached impressive values (20–75 µg/mL) in the mammary gland secretion of the four goats that developed gangrenous mastitis (Additional file 1).

### Expression of genes encoding mediators of immunity and inflammation at early stages post-infection

**Figure 8:**
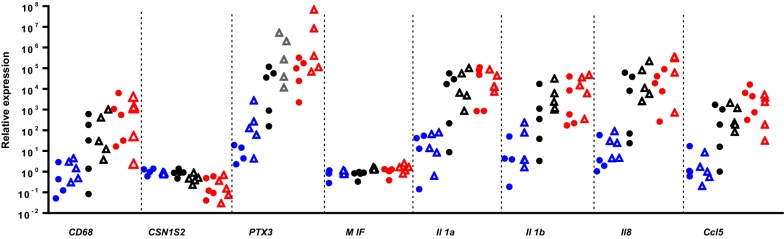
**Relative expression of genes encoding mediators of immunity and inflammation at early stages of infection using RNA extracted from MFG by Real-Time PCR.** The relative expression of a marker of contamination by leucocytes (CD68) and of genes involved in the inflammatory response of the mammary gland was monitored at 12 hpi (blue symbols), 18 hpi (black symbols) and 23 hpi (red symbols). Solid circles are for CI goats, hollow triangles for UI goats. Expression at 12, 18 and 23 hpi is relative to the expression (set at 1) at 0 hpi for each gene.

In an attempt to get information on the events that took place before 18 hpi, we measured the expression of a number of immunity mediators in MFG-associated RNA, which originates from mammary epithelial cells (MEC), by using RNA extracted form MFG and real time PCR. At that time, a few leucocytes had reached milk and mingled with the milk fat fraction, as confirmed by the increase in expression of the leucocyte marker CD68 (Figure 8). As soon as 12 hpi, there was a substantial increase in the relative expression of genes encoding the cytokines IL-1α and IL-1β, the chemokine IL-8, and the pentraxin PTX3 (Figure 8). There was a wide dispersion of values among animals, with no obvious distinctive trend between the UI and CI groups. Of note, there was no trend for a weaker response of goats of the UI group for *Il8* and *Ccl5* at 18 hpi, which is in accordance with the idea that the higher CFU concentrations at 18 hpi in the milk of the UI goats was not linked to a delayed response of the MEC.

**Figure 9:**
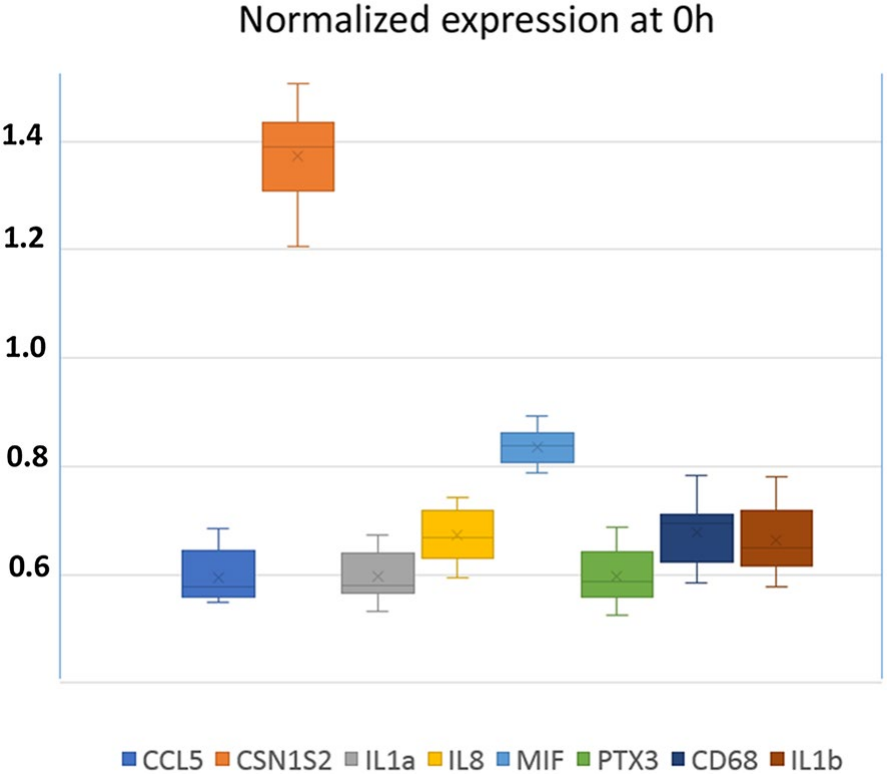
**Normalized expression of genes encoding mediators of immunity and inflammation at 0** **hpi.** RNA was extracted from MFG, just before inoculation (0 hpi) and submitted after reverse transcription to RT qPCR.

At 18 and 23 hpi, the relative expression of all the measured indicators rose sharply, except the expression of the genes encoding CSN1S2, which decreased slightly at 23 hpi, and MIF, which remained constant at 18 and 23 hpi (Figure 8). Interestingly, the cytokine MIF was highly constitutively expressed, second only to CSN1S2, compared to the less expressed cytokines and chemokines at 0 hpi (Figure 9). Noteworthy, transcripts of *Il17a* and *Il17f* were not found in the MFG-associated mRNA.

### Correlations and principal component analysis

A number of observations can be made from the Spearman’s rank correlation matrix (Table 4). There was a trend toward a negative correlation (r = −0.457; *p* = 0.096) between the clinical score and the SCC in the inoculated quarters at time of inoculation (0 hpi), suggesting that the initial SCC could relate to some resistance mechanism to infection. Nevertheless, the relation was still low at 12 hpi (−0.134). The positive and significant correlation between the clinical score and CFU numbers at 18 and 23 hpi reflects the importance of the bacterial burden that determines mastitis severity, and points to the necessity of an early control of bacterial proliferation. Of note, although there was a wide variation of CFU concentrations among animals at 12 hpi, this was of no consequence for the outcome of infection, since these concentrations did not correlate with the clinical score (r = 0.110, *p* = 0.379). This observation supports the idea that the decisive events took place between 12 and 18 hpi. With reference to the known importance of the phagocytic defense of the mammary gland by neutrophils, we could expect a correlation between the early increase in SCC and the outcome of infection. We have seen that at 12 hpi leucocyte recruitment to the mammary gland was just beginning (Figure 3). At 18 hpi, a time when the cell influx was full-blown, the SCC tended to correlate inversely with the clinical score (r = −0.549, *p* = 0.105), which suggests that cell concentration is of importance, but not determinant on its own. Another important parameter could be the efficiency of the phagocytic killing, since the ex vivo milk bactericidal activity at 18 hpi was highly inversely correlated with the clinical score (r = −0.859, *p* = 0.001), in keeping with the acknowledged importance of this effector mechanism for the control of infection. Accordingly, the phagocytic killing activity was highly inversely correlated with the milk CFU concentrations at 18 and 23 hpi. There was also a correlation between the phagocytic killing and the milk SCC at 18 hpi and at 23 hpi, in keeping with the role of the recruited leukocytes in the bactericidal activity. Of note, the SCC at 18 hpi correlated poorly with the milk bacterial concentrations at 23 hpi (r = −0.382, *p* = 0.279). It would have been expected that a higher SCC concentration at 18 hpi would correlate with a lower concentration of CFU at 23 hpi, as a result of phagocytic killing. This shows that the leukocyte concentration is not a robust predictor of the control of infection on its own, and that other parameters related to the efficiency of antibacterial agents (including phagocytes) are likely to play a determinant role. The inactivation of neutrophils by the leukotoxin LukMF′, as shown by cytospin slides examination (Figure 7) and the occurrence of toxic concentrations of LukMF′ in milk at 18 hpi, is likely to have contributed to the lack of control of the bacterial burden in the most severely affected goat.

**Table 4:** **Correlations between the investigated parameters (Spearman’s rank correlation)**

	Clinical score	CFU 12 hpi	CFU 18 hpi	CFU 23 hpi	Phago 18 hpi	SCC 0 hpi	SCC 12 hpi	SCC 18 hpi	SCC 23 hpi
Clinical score		0.110	**0.780**	**0.896**	**−0.859**	**−**0.457	**−**0.134	**−**0.549	**−**0.561
CFU 12 hpi	*0.379*		0.571	0.261	**−**0.187	**−**0.382	0.127	0.491	0.030
CFU 18 hpi	*0.005*	*0.044*		**0.894**	**−0.798**	**−**0.328	0.006	**−**0.121	**−**0.468
CFU 23 hpi	*0.0004*	*0.235*	*0.0005*		**−0.899**	**−**0.224	0.042	**−**0.382	**−**0.588
Phago 18 hpi	*0.001*	*0.304*	*0.004*	*0.0004*		0.200	0.136	0.562	0.679
SCC 0 hpi	*0.096*	*0.139*	*0.174*	*0.268*	*0.292*		0.2	−0.042	0.345
SCC 12 hpi	*0.353*	*0.366*	*0.5*	*0.459*	*0.353*	*0.292*		0.309	0.321
SCC 18 hpi	*0.052*	*0.077*	*0.366*	*0.139*	*0.048*	*0.459*	*0.193*		0.430
SCC 23 hpi	*0.048*	*0.473*	*0.089*	*0.040*	*0.017*	*0.165*	*0.184*	*0.109*	

Numbers in the higher half are correlations, in the lower half and in italics the *p* values. Correlations indicated in bold figures are highly significant (*p* ≤ 0.005).

**Figure 10:**
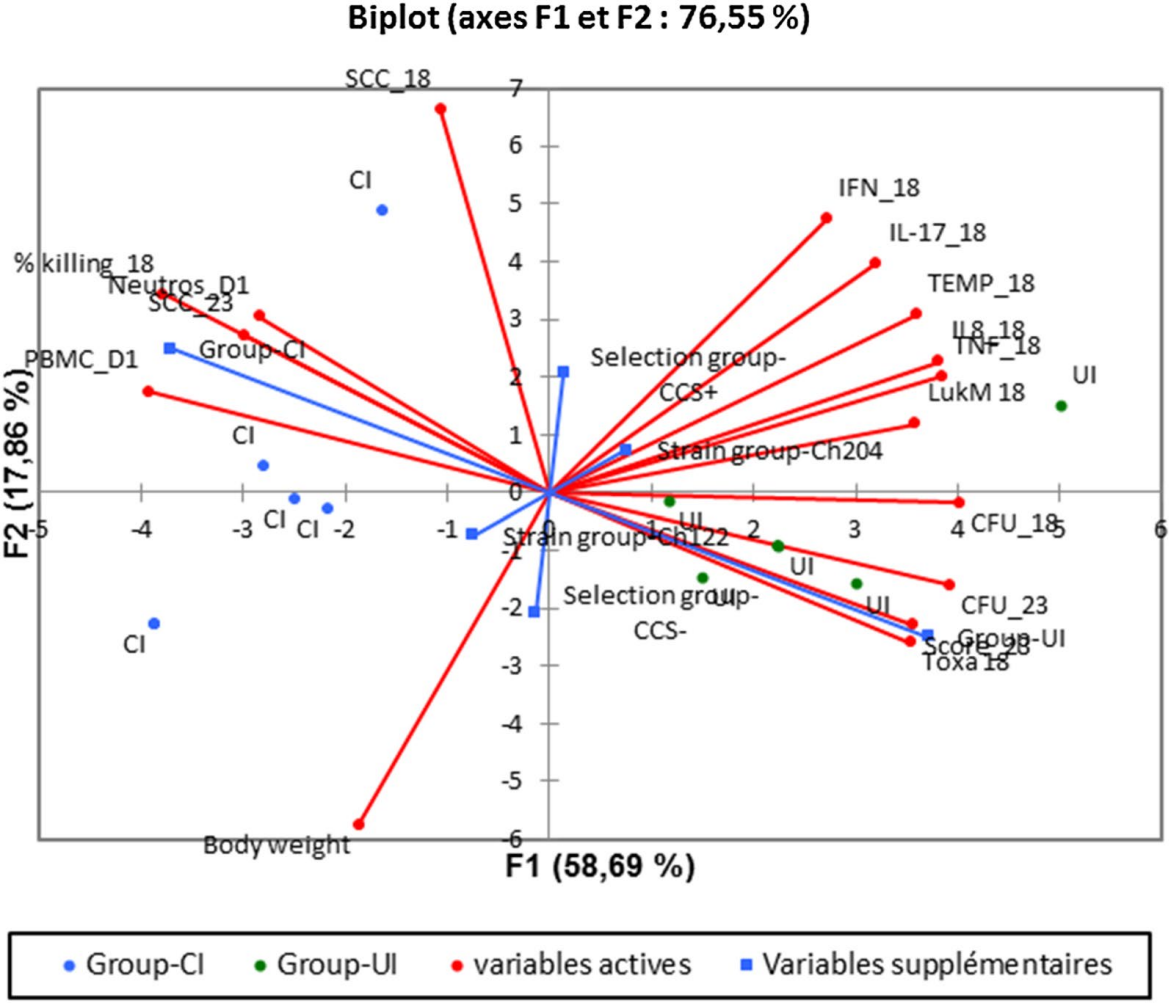
**Biplot representation of the principal component analysis of the clinical, infection and inflammation parameters variability.** The 16 variables included were: body weight before infection, body temperature at 18 hpi, clinical score at 23 hpi, PBMC and blood neutrophil concentrations at 23 hpi, milk SCC at 18 and 23 hpi, milk CFU concentrations at 18 and 23 hpi, % killing by milk ex vivo at 18 hpi, a-toxin and LukM milk concentrations at 18 hpi, and IFN-γ, IL-17A, TNF-α and IL-8 milk concentrations at 18 hpi. Supplementary variables are the selection groups (CCS+ and CCS−), the infecting *S. aureus* strains (Ch122 or Ch204), and the severity groups (uncontrolled infection UI or controlled infection CI). Blue dots are the five CI goats, green dots the five UI goats. Analysis was performed with the XLStat software (Spearman correlation, distance biplot).

The principal component analysis biplot based on 16 variables clearly discriminates UI from CI goats. Dimension one correlates mainly with the severity of mastitis, with the clinical score, bacterial burden, bacterial toxins and inflammatory cytokines on the side of the UI group, and with cell concentrations in blood and milk and killing efficiency on the side of the CI group (Figure 10). The 18 hpi SCC and body weight correlated poorly with the first dimension. It was apparent from the biplot that both the selection group and the inoculated strain were not linked to the measured variables.

## Discussion

Initially, the study was devised as an experiment intended to determine the best experimental design capable of uncovering differences in the response of two SCS selection goat lines to staphylococcal infection. In fact, a previous attempt based on experimental infections of 10 goats had not revealed different responses to *S. aureus* between the two lines [10]. This result is in contrast with the data obtained under natural conditions of infection, which showed that low SCS goats had significantly lower udder infection rate than high SCS goats [5]. The discrepancy may come from the low number of animals studied in the experimental infection studies and the fact the genetic selection is not associated with full resistance but improved udder health with a level of variability between animals. The genetic selection on SCS may also involve the selection of passive defense mechanisms (e.g. udder and teat anatomy or physiology) that are by-passed by experimental infections. It appeared that the infections caused by the two strains used in our study were of higher severity than those caused by the strain used in the previous study in Italy, which elicited lower SCC peak values without gangrenous evolution. This strain reportedly did not possess the genes encoding the leukotoxin LukMF′, which could contribute to a lower pathogenicity, and there was no indication as to the production of α-toxin. Yet, our result was unanticipated, because previous experimental goat infections with the supposed most virulent of the two strains tested (Ch122) had seldom induced very severe mastitis ([11] and P. Rainard, unpublished results). The reason behind the higher severity of infection in this study could be related to the very low number of cells in the milk of the challenged goats at time of inoculation (< 50 000 cells/mL), compared to mean cells numbers > 450 000/mL in the previous experiment with selected goats [10].

Another source of inconsistency across the published and the present studies is the natural variability associated with experimental results, especially when the samples are small. Fortuitous associations can be found, and the conclusions that we tend to draw from the results are not necessarily valid. This for example applies to the attribution to *S. aureus* strains of different virulence potentials on the basis of either isolation from clinical or subclinical mastitis cases, which is clearly spurious at the individual strain level, or even of a limited number of experimental infections. This is exemplified in our studies that used the same *S. aureus* strain with very different clinical outcomes. On the other hand, this is an indication that the strain is not all that counts, but that chance and other concomitant factors, in particular the host physiological and immune status at time of infection, are also important players. Hence, the idea that host factors determine the evolution of infection to gangrenous mastitis. In addition, correlations must be considered with caution, because they can result from chance rather than causation. Nevertheless, the correlations that we considered in our study involved mainly variables that are usually thought of as causally related, such as concentrations of milk cells (mainly neutrophils), milk cfus and phagocytic efficiency.

Importantly, there was no apparent link between the outcome of infection and either the infecting strain or the SCS-based selection group. Five goats overcame the infection and avoided severe mastitis, the other five goats did not, four of them developing a gangrenous mastitis, the fifth one finally failing to control the bacterial burden. Three of the five severely affected goats had received the anticipated less virulent strain, and the four gangrenous cases fell evenly in the two selection groups. This unanticipated result oriented the analysis of the data towards the description of individual trajectories and the search for possible explanation for the different degrees of severity of infections experienced by the challenged goats.

The most salient difference between the two severity groups was the milk concentration of staphylococci at 18 hpi, which clearly segregated the UI from the CI goats (Figure 5). Other dividing factors were the occurrence of high concentrations of α-toxin and LukMF′ in the milk of the UI goats, and the inefficiency of the killing activity of their 18 hpi-milk (Figure 6). These events are likely causally related to the inactivation of the phagocytic capacity of neutrophils by the leukotoxin LukMF′ [26, 27]. Cytotoxic concentrations of this leukotoxin were found in milk in the presence of specific antibodies, which reportedly do not preclude the detection of leukotoxins in the milk of infected mammary gland [28]. This was supported by the negative correlation (−0.899) between the 18 hpi milk phagocytic activity and the CFU numbers at 23 hpi (Table 4). Of note, the outcome of infection was not related to the concentration of cells in 18 hpi milk, i.e. did not result from a differential leucocyte recruitment among the goats. This again supports the importance of the phagocytic capacity of the recruited neutrophils.

In accordance with the similar initial leucocyte recruitment of leukocytes, there were comparable concentrations of neutrophil-attracting chemokines (IL-8 and CXCL3) in the first 23 hpi (Figure 3). This corroborated with similar qPCR results for *Il8* and *Ccl5* at 12 and 18 hpi between UI and CI goats (Figure 8). Milk concentrations of TNF-α and IFN-γ, cytokines that can stimulate the phagocytic killing capacity of neutrophils [29, 30], were higher in the milk of the most severely affected goats (Figure 4). This was probably the consequence of the stronger immune stimulation by the higher bacterial load, but did not prevent the loss of net phagocytic killing observed in the 18 hpi milk. The cytokine IL-17A is able to stimulate the pro-inflammatory and anti-microbial activities of mammary epithelial cells through the production of chemokines and antimicrobial peptides, especially in the presence of staphylococcal agonists of the innate immune system [31]. Interestingly, there was an early increase in IL-17A concentration at 18 hpi in the milk of almost all goats (Figure 4C), suggesting that IL-17A induction is a component of the immune response of the goat mammary gland to infection by *S. aureus*. Transcripts of *Il17a* and *Il17f* were not found in the MFG-associated RNA at 18 and 23 hpi, suggesting that milk IL-17A did not originate from MEC or milk fat-associated CD68+ leukocytes (mainly myeloid cells). Concentrations of IL-17A sky-rocketed in the secretion of the gangrenous glands, partly as a result of the reduction in secretion and exudate volume, but indicating also that there was a strong stimulus for IL-17A production by cells that remain to be identified. Another cytokine that is likely to contribute to the defense of the mammary gland is IL-22, which is endowed with epithelium stimulatory and healing properties [32]. Concentrations of IL-22 rose in milk after inoculation, without an initial peak at 18 hpi, but increased sharply in the mammary gland secretion when symptoms worsened (Figure 4D). A somewhat protracted secretion of IL-22 in the milk of cows infected with *Escherichia coli* has been reported [21]. At first sight, the production of IL-22 was not determinant for the outcome of infection. As for TNF-α, the IL-17A and IL-22 increases were likely related to the magnitude of the bacterial burden and stimulus, and they were apparently unable to improve the course of infection. Of note, concentrations of IFN-γ were often hardly detectable, and remained low except in the secretion of a few goats when compared to IL-17A and IL-22 concentrations (Figure 4C and D). The expression of two other genes encoding immune defense components were monitored during the first phase of infection. The expression of the multifunctional cytokine MIF appeared to be constitutive considering its relatively high expression at 0 hpi and the absence of overexpression at 18 and 23 hpi. Its expression in the 0 and 12 hpi MFG-associated RNA strongly suggests that MIF is expressed constitutively by MEC, which is consistent with its reported constitutive expression by epithelial linings [33]. MIF is present in human milk, in particular in the cytoplasmic component in the MFG fraction, which supports its production by MEC [34]. This cytokine has been implicated in wound healing and epithelium repair [35]. The pentraxin PTX3, which is produced by several innate immunity cell types, plays essential roles in the innate immune defense against infections [36]. PTX3 has been found in bovine, ovine and caprine mastitis milk and the expression of its encoding gene by MEC is increased in response to infection by mastitis-associated pathogens [10, 24, 37, 38]. In our study, *Ptx3* overexpression was obvious at 12 hpi and was considerable at 18 and 23 hpi, in particular in MFG (hence in MEC) of UI goats (Figure 8). This probably reflected a more intense stimulus as mirrored by TNF-α milk concentrations.

A noticeable find of this study was the high concentrations of α-toxin in the milk of goats that developed gangrenous mastitis. Toxin-producing strains of *S. aureus* are the most common cause of gangrenous mastitis in dairy ruminants [39–41]. Mastitis-associated strains of bovine origin belonging to certain lineages such as the sequence type ST151 are known to highly express genes encoding cytotoxins such as α-toxin and LukMF′ [42]. The role of α-toxin is considered of prime importance for the evolution towards the gangrenous form of mastitis based on its capacity to cause arteriole vasoconstriction and subsequent ischemic necrosis. Injection of *S. aureus* culture supernatant containing α-toxin into the mammary gland of mice induces a coagulative necrosis [43]. A feature of bovine mastitis *S. aureus* isolates is the in vitro production of impressive amounts of α-toxin when compared to the production by human clinical *S. aureus* isolates, resulting from both genomic and transcriptional peculiarities [44]. Our results extend this finding to the in vivo production of α-toxin by two caprine mastitis strains in the course of mammary gland infection, with concentrations in the range of 50–75 µg/mL in mammary gland secretions. Antibodies to α-toxin are supposed to play an important role in the protection from severe forms of staphylococcal mastitis, as ewes with high titers of antibodies to α-toxin did not develop gangrenous mastitis [45]. In our study, all goats had rather low titers of antibodies to α-toxin and LukM, which proved to be insufficient to neutralize the massive toxin production that occurred when bacterial multiplication got out of control.

**Figure 11:**
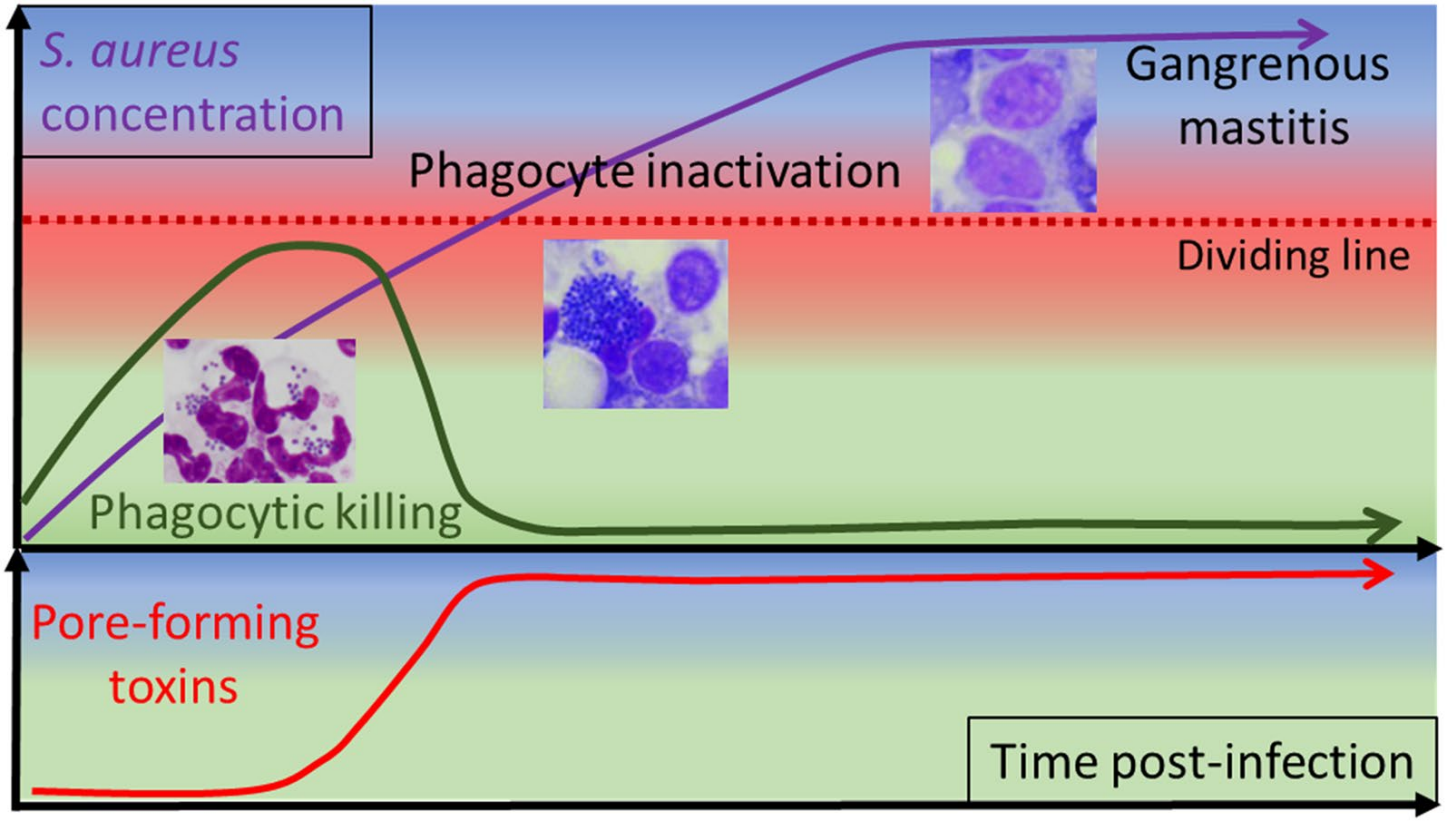
**Schematic evolution of infection towards gangrenous mastitis.** The purple line represents the evolution with time of the bacterial concentration, and also symbolizes the evolution towards gangrenous mastitis, which is tightly linked to the bacterial concentration. High concentration allows *S. aureus* to produce high amounts of pore-forming toxins (red line), among which leukotoxins inhibit the phagocytic killing (green line) by the phagocytes recruited in milk, whereas α-toxin contributes to the ischemic necrosis that results in gangrenous mastitis. The dividing line corresponds to the critical bacterial concentration that allows bacteria to overwhelm the recruited phagocytes, and to impede phagocytic killing. The microphotographs show (from left to right) images of *S. aureus* phagocytosed by neutrophils, rafts of staphylococci and intoxicated neutrophils, and the typical appearance of neutrophils intoxicated by LukMF′ (with swollen and rounded nucleus).

In conclusion, this study led us to propose a few explanatory factors for the evolution of *S. aureus* mammary gland infection towards the gangrenous form. The determining parameter was the concentration of bacteria in milk, and most likely their absolute numbers, taking into account milk volume, which remained high in the first phase of the infection. Exotoxins, in particular the pore-forming toxins, were produced in toxic amounts. These toxins, such as the α-toxin and the leukotoxins are partly under the control of quorum sensing through different regulators and are produced when *S. aureus* concentration is sufficient [44, 46]. In our study, about 10^7^ CFU/mL were necessary for the production of LukMF′ at concentrations capable of inactivating caprine neutrophils. Accordingly, phagocytic killing was severely impaired and the infection evolved towards very severe, even gangrenous form. The ischemic necrosis which characterizes gangrenous mastitis is likely due to the vasoconstriction action of α-toxin on the arterioles that irrigate the mammary tissue, occasioning tissue death [47]. In our study, high concentrations of α-toxin were found in the milk of the goats that developed gangrenous mastitis. This abundant production of α-toxin, concomitant with a heavy bacterial burden and the inactivation of phagocytes, was probably instrumental in provoking the local ischemic necrosis leading to mammary gland gangrene. Schematically, the dividing line between the evolution towards gangrenous mastitis or towards the control of infection would be set by the concentration of bacteria (Figure 11). In our experimental model, the line was crossed by 18 hpi. As the production of the two toxins monitored in our study may be considered a terminal event, because depending of a rather high bacterial concentration, other staphylococcal virulence factors and early host defenses were of major importance. The precise phenomena behind the segregation of the two severity groups of goats, which took place between 12 and 18 hpi, remain to be uncovered.

## Additional file

**Additional file 1.** **Antibody titers to the leukotoxin component LukM and α-toxin and concentration of α-toxin or LukM in milk.** Antibody titers to LukM and α-toxin in serum and milk were calculated with reference to an immune goat serum arbitrarily given 1000 or 200 antibody units, respectively. Concentrations of LukM in mammary gland secretions were determined by sandwich ELISA, and of α-toxin by competitive ELISA.

## Abbreviations

CCS: somatic cell count
CD: cluster of differentiation
CFU: colony-forming unit
CSN1S2: casein 1S2
CXCL: chemokine (C-X-C motif) ligand
DPBS: Dulbecco’s phosphate buffered saline
ELISA: Enzyme-Linked Immunosorbent assay
hpi: hour post-inoculation
IFN-γ: interferon- γ
IL: interleukin
Luk: leukotoxin
MEC: mammary epithelial cell
MFG: milk fat globules
MIF: migration inhibitory factor
PCA: principal component analysis
PPIA: peptidylprolyl isomerase A
PTX3: pentraxin 3
RPS24: ribosomal protein S24
SCS: somatic cells score
TNF-α: tumor necrosis factor-α
